# RNA-Binding Protein MSI2 Binds to miR-301a-3p and Facilitates Its Distribution in Mitochondria of Endothelial Cells

**DOI:** 10.3389/fmolb.2020.609828

**Published:** 2021-01-21

**Authors:** Qian Qian Guo, Jing Gao, Xiao Wei Wang, Xian Lun Yin, Shu Cui Zhang, Xue Li, Lian Li Chi, Xiao Ming Zhou, Zhe Wang, Qun Ye Zhang

**Affiliations:** ^1^The Key Laboratory of Cardiovascular Remodeling and Function Research, Chinese Ministry of Education and Chinese National Health Commission, Shandong University, Jinan, China; ^2^Department of Cardiology, Cheeloo College of Medicine, Qilu Hospital, Shandong University, Jinan, China; ^3^The State and Shandong Province Joint Key Laboratory of Translational Cardiovascular Medicine, Jinan, China; ^4^Cardiovascular Disease Research Center of Shandong First Medical University, Central Hospital Affiliated to Shandong First Medical University, Jinan, China; ^5^National Glycoengineering Research Center, Shandong University, Qingdao, China; ^6^Institute of Endocrinology, Shandong Academy of Clinical Medicine, Jinan, China; ^7^Shandong Clinical Medical Center of Endocrinology and Metabolism, Jinan, China; ^8^Division of Endocrinology and Metabolism, Shandong Provincial Hospital Affiliated to Shandong University, Jinan, China; ^9^Division of Geriatrics, Shandong Provincial Hospital Affiliated to Shandong University, Jinan, China

**Keywords:** mitochondrial miRNA distribution, RNA-binding protein, Musashi RNA binding protein 2, reactive oxygen species, endothelial cell injury

## Abstract

Numerous miRNAs have been detected in mitochondria, which play important roles in many physiological and pathophysiological processes. However, the dynamic changes of miRNA distribution in mitochondria and their mechanisms in reactive oxygen species (ROS)-induced endothelial injury remain unclear. Therefore, miRNA levels in whole cells and mitochondria of H_2_O_2_-treated endothelial cells were analyzed by small RNA sequencing in the present study. The results showed that H_2_O_2_ significantly reduced the relative mitochondrial distribution of dozens of miRNAs in human umbilical vein endothelial cells (HUVECs). Among the high-abundance miRNAs, miR-301a-3p has the most significant changes in the redistribution between cytosol and mitochondria confirmed by absolute quantitative polymerase chain reaction (qPCR). To unravel the mechanism of miR-301a-3p distribution in mitochondria, RNA pull-down followed by label-free quantitative proteomic analysis was performed, and RNA-binding protein Musashi RNA binding protein 2 (MSI2) was found to specifically bind to miR-301a-3p. Western blotting and immunofluorescence colocalization assay showed that MSI2 was located in mitochondria of various cell types. H_2_O_2_ significantly downregulated MSI2 expression in whole endothelial cells, promoted the distribution of MSI2 in cytosol and decreased its distribution in the mitochondria. Moreover, overexpression of MSI2 increased the mitochondrial distribution of miR-301a-3p, whereas inhibition of MSI2 decreased its distribution in mitochondria. Thus, MSI2 might be responsible for the distribution of miR-301a-3p between cytosol and mitochondria in endothelial cells. Our findings revealed for the first time that MSI2 was involved in the regulation of miRNA distribution in mitochondria and provided valuable insight into the mechanism of mitochondrial distribution of miRNAs.

## Introduction

It is believed that miRNAs regulate mRNA stability and translation primarily in the cytosol (Gebert and MacRae, 2019). However, a growing body of evidence indicated that miRNAs are also distributed in various subcellular regions, such as nucleus, processing bodies, endoplasmic reticulum and mitochondria (Leung, 2015; Trabucchi and Mategot, 2019). Subcellular distribution of miRNAs is indicative of regulatory role of miRNA in various cellular processes and it profoundly influenced many pathophysiological processes (Trabucchi and Mategot, 2019). To date, hundreds of mature miRNAs have been detected in mitochondria of various types of cells and tissues (Barrey et al., 2011; Sripada et al., 2012a). Many miRNAs located in mitochondria have been found to regulate many general and mitochondrial-specific biological processes (Borralho et al., 2014). Moreover, the expression profile of miRNAs in mitochondria was dynamically changed under different pathophysiological conditions (Wang et al., 2015).

It is well-known that the genome of human mitochondria only encodes 13 proteins, 22 tRNAs, and 2 rRNAs (Anderson et al., 1981). Numerous nucleus-encoded proteins and ncRNAs must be imported into mitochondria via sophisticated transport systems to maintain proper mitochondrial function. To date, several proteins have been found to contribute to the transport of nucleus-encoded non-coding RNAs (ncRNAs) including tRNAs, rRNA, and RNA component of mitochondrial RNA processing endoribonuclease (MRP RNA) into mitochondria (Kim et al., 2017). The polynucleotide phosphorylase (PNPase) is also associated with the import of miRNA-378 into mitochondria (Shepherd et al., 2017). Considering the complexity of mitochondrial miRNAs and their transport system, it should be a reasonable assumption that many unknown players apart from PNPase must be involved in the mitochondrial distribution of miRNAs. However, the mechanisms regulating the distribution of miRNAs in mitochondria are still poorly understood.

In the present study, we found that H_2_O_2_ noticeably reduced the levels of many miRNAs in mitochondria. In particular, the redistribution of miR-301a-3p between the cytosol and mitochondria was further confirmed by absolute quantitative polymerase chain reaction (qPCR). Moreover, our results demonstrated that Musashi RNA Binding Protein 2 (MSI2) could specifically bind to miR-301a-3p and facilitate its distribution in mitochondria. MSI2 downregulation may be responsible for the redistribution of miR-301a-3p in mitochondria of H_2_O_2_-treated endothelial cells.

## Materials and Methods

### Cell Culture and Transfection

Human umbilical vein endothelial cells (HUVECs) were cultured in complete endothelial cell medium (#1001, ScienCell, Carlsbad, CA, USA) supplemented with 5% fetal bovine serum (FBS), 100 U/mL penicillin, 100 μg/mL streptomycin, and 1% endothelial cell growth supplement (ECGS) at 37°C in a humid atmosphere with 5% CO_2_. HepG2 cells and HEK293T cells were cultured in Dulbecco's modified Eagle's medium (#0030034DJ, Gibco, Rockville, MD, USA) containing 10% FBS (#04-001-1A, Biological Industries, Kibbutz Beit Haemek, Israel), 100 U/mL penicillin, and 100 μg/mL streptomycin (#15070063, Gibco). Human arterial smooth muscle cells (HASMCs) were cultured in smooth muscle cell medium (SMCM), 1% SMCM growth supplements, 2% FBS, 100 U/mL penicillin, and 100 μg/mL streptomycin (#1101, ScienCell). The siRNAs targeting MSI2 gene were purchased from Ribobio (Guangzhou, China) and transfected at 50 nM using Lipofectamine RNAiMAX reagent (#13778030, Life Technologies, Carlsbad, CA, USA). The pCMV3-MSI2 expression plasmid was purchased from Sino Biological (#HG13069-NF, Beijing, China). Plasmid transfection was performed using Lipofectamine 3000 reagent (#L3000001, Life Technologies).

### Subcellular Fractionation

The mitochondrial and cytosolic fractions of HUVECs were isolated as described previously (Clayton and Shadel, 2014a,b). First, the cell pellet was washed twice in cold phosphate-buffered saline (PBS) and then resuspended in hypotonic buffer. Next, the cell suspensions were homogenized by a motor-driven Potter-Elvehjem homogenizer followed by centrifugation twice to collect the debris and nuclei. To collect the crude mitochondria, the supernatants were further centrifuged at 15,000 × *g* for 15 min at 4°C. The supernatants were collected as cytosolic fractions. Next, the crude mitochondria were layered on a discontinuous sucrose gradient (1.0M and 1.5M) and ultra-centrifugated at 60,000 × *g* for 40 min at 4°C. Then, the purified mitochondria were obtained from the interface between the two sucrose cushions. Next, the purified mitochondria were treated with RNase I (#AM2295, Life Technologies) to decontaminate the cytosolic RNA, followed by stopping the activity of RNase I using a ribonuclease inhibitor (RNasin, #N2615, Promega, Madison, WI, USA). The final mitochondrial pellet was resuspended in 100 μL RNAlater (#AM7020, Life Technologies) for miRNA quantitative real-time polymerase chain reaction (qRT-PCR) analysis and small RNA sequencing.

### Immunoprecipitation and Western Blotting

For immunoprecipitation, purified mitochondria from HUVECs were lysed in NP-40 buffer on ice for 20 min followed by centrifugation at 12,000 × *g* for 25 min. The mitochondrial lysate was then incubated with 2 μg anti-MSI2 antibody (#10770-1-AP, Proteintech, Wuhan, China) or anti-AGO2 antibody (#10686-1-AP, Proteintech) and 40 μL Protein A/G PLUS-Agarose beads (#sc-2003, Santa Cruz Biotechnology, Dallas, Texas, USA) overnight at 4°C with gentle rotation. The beads were washed and boiled in 2X SDS polyacrylamide gel electrophoresis (SDS-PAGE) loading buffer. Non-immune IgG (#12-370, Millipore, Burlington, MA, USA) was used as a negative control. For western blotting, total and mitochondrial proteins were extracted and loaded on a 10% gel followed by gel electrophoresis. Subsequently, the separated proteins were transferred to a 0.2 μm polyvinylidene difluoride membrane (#1620177, Bio-Rad, Berkeley, CA, USA). Next, the membrane was blocked with 5% non-fat milk and incubated with primary antibodies overnight at 4°C with gentle rotation. After washing, the membrane was incubated with the corresponding secondary antibodies at room temperature for 1 h followed by visualization using a ChemiDoc XRS+ imaging system (Bio-Rad). All antibodies used in this study were listed in Supplementary Table 1.

### Quantitative Real-Time PCR (qRT-PCR)

Total RNA in the whole cell, mitochondrial fraction, or cytosolic fraction was extracted using TRIzol reagent (#15596026, Life Technologies). To evaluate mRNA expression levels, cDNA synthesis and PCR amplification were performed using PrimeScript RT Master Mix and TB Green Premix Ex Taq II (#RR036A, RR820B, Takara, Kusatsu, Japan). The miR-301a-3p primer was purchased from Takara and its levels were determined using the Mir-X miRNA First-Strand Synthesis Kit and Mir-X miRNA qRT-PCR TB Green Kit (#638313, 638314, Clotech, Kusatsu, Japan) according to the manufacturer's instructions. MiRNA levels in the mitochondria were normalized to those of 12S rRNA. To absolutely quantify the amount of miR-301a-3p, a serial dilution of synthetic miR-301a-3p single-stranded RNA oligonucleotides (Ribobio) was used to generate the standard curve by qRT-PCR. The primers for mRNAs and miRNAs used in our study were listed in Supplementary Table 2.

### Small RNA Sequencing and Data Analysis

Small RNA sequencing was performed on an Illumina Hiseq X Ten (Illumina, San Diego, CA, USA; SE50 model) according to the manufacturer's instructions. After removing adapters and filtering low-quality reads, the high-quality clean data were mapped to the human genome sequence (hg19) and the unmapped reads were filtered. Then the mapped reads were aligned to the miRNA database using the R package bowtie2 (version 2.0.6). The sequencing data have been deposited in ArrayExpress (E-MTAB-9851).

### Mitotracker and Immunofluorescence Staining

Cells were treated with MitoTracker Red CMXRos (#M7512, Thermo Fisher, Waltham, MA, USA) to label mitochondria according to the manufacturer's instructions. After fixation and permeabilization, the cells were successively incubated with anti-MSI2 antibody (#10770-1-AP, Proteintech) and Alexa Fluor 488-conjugated secondary antibody (#SA00013-2, Proteintech). The nuclei were stained using 4',6-diamidino-2-phenylindole (DAPI, #10236276001, Sigma, China). Immunofluorescent images were obtained using a Zeiss LSM 710 confocal microscope (Zeiss, Oberkochen, Germany) and colocalization analysis was performed using Image-Pro Plus 6.0 software (Media Cybernetics, Rockville, MD, USA).

### RNA Immunoprecipitation

RNA immunoprecipitation was performed using the Magna RIP RNA-Binding Protein Immunoprecipitation Kit (#17-700, Millipore) following the manufacturer's instructions. Briefly, HUVECs were lysed in the ice-cold lysis buffer supplemented with protease inhibitors and recombinant RNase inhibitors. Five micrograms of anti-MSI2 antibody (#10770-1-AP, Proteintech) or control Rabbit IgG (#12-370, Millipore) and 50 μl magnetic beads were incubated in 0.5 ml wash buffer for 30 min to prepare magnetic beads for immunoprecipitation. Then prepared magnetic beads with 100 μl cell lysate were suspended in 900 μl immunoprecipitation buffer to immunoprecipitate RNA-binding protein-RNA complexes. After rotation at 4°C overnight, the beads containing RNA-binding protein-RNA complexes were washed total six times with 500 μL of cold wash buffer. Then the proteinase K buffer was added into the tube and incubated at 55°C for 30 min with shaking to digest the protein, followed by the purification of RNA which was carried out by phenol, chloroform, Salt Solution I, Salt Solution II, Precipitate Enhancer and absolute ethanol. Finally, coimmunoprecipitated miRNAs were determined using the Mir-X miRNA First-Strand Synthesis Kit and Mir-X miRNA qRT-PCR TB Green® Kit (#638313, 638314, Clotech).

### Biotinylated miRNA Pull-Down Assay

HUVECs were collected and incubated with 100 μl hypotonic buffer (10 mM Tris-Cl [pH 7.5], 20 mM KCl, 1.5 mM MgCl_2_, 5 mM DTT, 0.5 mM EGTA, 5% glycerol, 0.5% NP-40) supplemented with RNase inhibitors (#N2615, Promega) and protease inhibitors (#5892970001, Roche Applied Science, Penzberg, Germany) on ice for 5–10 min. Then, a homogenizer was used to mechanically disrupt the cell membrane. The cell homogenization was centrifuged at 2,000 × *g* at 4°C for 10 min to remove the nucleus, and then at 10,000 × *g* for 10 min at 4°C to further purify the supernatant. The protein concentration was determined using the BCA method. Then, 6 μg of biotinylated RNA oligonucleotides (~1 nM) in 200 μl high-salt wash buffer (HS-WB: 20 mM HEPES, pH 8.0, 300 mM KCl, 10 mM MgCl_2_, 0.01% NP-40, 1 mM DTT) was bound to 20 μl streptavidin–agarose beads (#S1638, Millipore), previously blocked with 1 mg/mL yeast tRNA (#10109495001, Roche Applied Science), during a 3 h incubation at 4°C on a turning wheel. Then, streptavidin–agarose beads carrying 6 μg of oligonucleotides were collected and washed three times in HS-WB buffer followed by incubation with 100 μl cell lysate supernatant on a slowly rotating turning wheel for 30 min at room temperature, and then at 4°C for 2 h. The streptavidin–agarose beads were then washed four times with HS-WB containing increasing amounts of KCl (0.6, 0.8, 1.2, and 2 M) to reduce background noise. The specifically bound proteins were eluted with 100 μl of 6 M urea with gentle shaking at room temperature for 30 min. The protein concentration of the elution was then determined by the Bradford method and the elution was freeze-dried for preservation until MS analysis was performed. The biotinylated RNA oligonucleotides used in the present study were shown in Supplementary Table 3.

### Label-Free Quantitative (LFQ) Proteomic Analysis

Protein samples were prepared for label-free quantitative (LFQ) proteomic analysis by nano-liquid chromatography-tandem mass spectrometry (LC-MS/MS) using the filter-aided sample preparation (FASP) protocol as previously described with minor modifications (Wiśniewski et al., 2009). Briefly, after reduction and alkylation, the protein samples were transferred to Microcon YM-10 filter units (#1602002vs, Sartorius Stedim Biotech, Göttingen, Germany) and centrifuged to remove detergent. Then, they were digested by 1:25 (w/w) trypsin (#T2600000, Sigma) at 37°C overnight. MS/MS analysis was performed on an EASY-nLC 1000 liquid chromatograph coupled with a Q Exactive Plus mass spectrometer (Thermo Scientific). Raw MS data were analyzed using Proteome Discoverer software 2.2 (Thermo Scientific) for protein identification and quantification according to the manual of this software. The detailed procedure was described in the Supplemental Methods.

### Bio-Layer Interferometry (BLI)

ForteBio Octet RED 96 (Forte Bio, Fremont, CA, USA) was used to perform the BLI assay. Briefly, streptavidin-coated biosensors (Forte Bio) were bound to the biotinylated RNA oligonucleotides and incubated with protein samples at different concentrations to measure the binding kinetics between the MSI2 protein and biotinylated RNA oligonucleotides. The detailed procedure was described in the Supplemental Methods.

### Annexin V/Propidium Iodide and EdU Staining

The apoptosis of HUVECs treated or untreated with hydrogen peroxide (H_2_O_2_) was evaluated using the Annexin V/propidium iodide apoptosis detection kit (#556547, BD Pharmingen, San Jose, CA, USA) following the manufacturer's protocol. Briefly, HUVECs were seeded on 6-wells plates and treated with 100 μM H_2_O_2_. After treatment with H_2_O_2_, HUVECs were washed twice with ice-cold PBS and trypsinized to detach cells. Then the cells were resuspended and 5 μl FITC Annexin V with 5 μl propidium iodide were added. After incubating for 15 min at room temperature in the dark, the cells were analyzed by flow cytometry. In addition, an EdU staining assay (#ab219801, Abcam) was performed to assess cell proliferation. Briefly, after treatment with H_2_O_2_, 20 mM 5-ethynyl-20 -deoxyuridine (EdU) was added to the culture medium and incubated for 6 h at 37°C in the dark. The cells were then fixed with 4% paraformaldehyde (PFA), washed with 3% bovine serum albumin (BSA) and permeabilized using 0.5% Triton X-100. Then, EdU reaction cocktail, which was prepared according to the manufacturer's procedure was added and incubated for 30 min. Flow cytometry was used to analyze EdU staining.

### MTT Assay

The effect of glucose oxidase (GO) on endothelial cell viability was assessed by MTT assay. After treating HUVECs with different concentrations of GO for 24 h, MTT solution (5 mg/mL, #C0009S, Beyotime, Shanghai, China) was added to the culture medium and incubated for a further 4 h to generate an insoluble formazan crystal. Then the formazan crystal was solubilized in dimethyl sulfoxide (DMSO, #D8418, Sigma). The absorbance was measured at 570 nm in a microplate reader (SynergyH1 Hybrid Multi-Mode Reader, Biotek, Winooski, VT, USA).

### Statistical Analysis

Data normality was evaluated using Shapiro–Wilk test. The Wilcoxon rank-sum test was used for the comparison of two-group of non-normally distributed data. Two-tailed Student's *t*-tests and one-way analysis of variance (ANOVA) were used for comparison of two-group and multiple-group of normally distributed data, respectively. Statistical analysis was performed using GraphPad Prism v8.1. *P* < 0.05 was considered statistically significant. All experiments were repeated independently at least three times.

## Results

### ROS Leads to miR-301a-3p Redistribution Between Cytosol and Mitochondria in HUVECs

**Figure 1:**
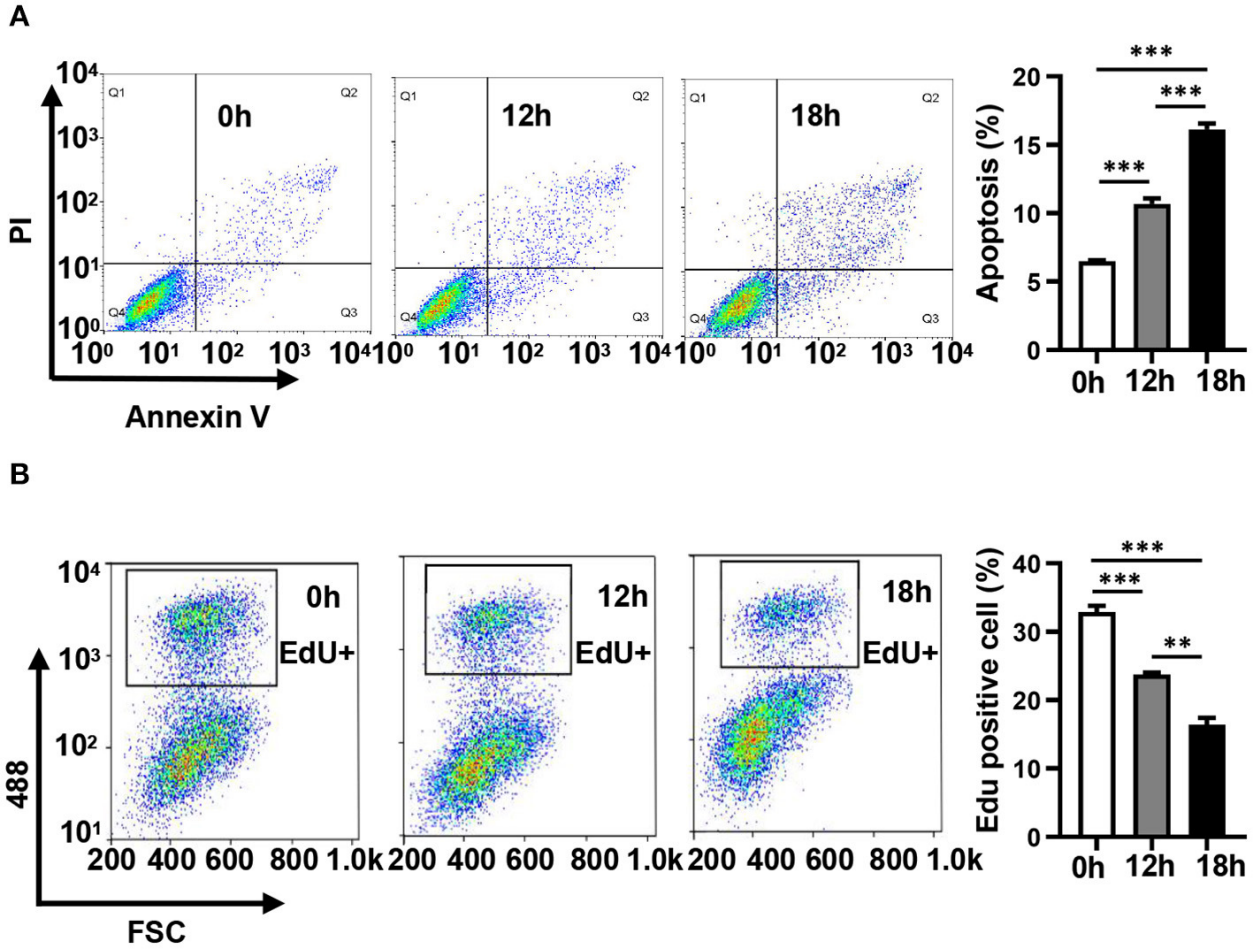
Hydrogen peroxide induces apoptosis and inhibits the proliferation of HUVECs. **(A)** The apoptosis of HUVECs treated with 100 μM H_2_O_2_ for 0, 12, and 18 h was detected by Annexin V/ propidium iodide staining. **(B)** The proliferation of HUVECs treated with 100 μM H_2_O_2_ for 0, 12, and 18 h was detected by EdU-iFluor488. One-way analysis of variance (ANOVA) was used for statistical analysis. Data are presented as mean ± SEM. **P < 0.01; ***P < 0.001.

**Figure 2:**
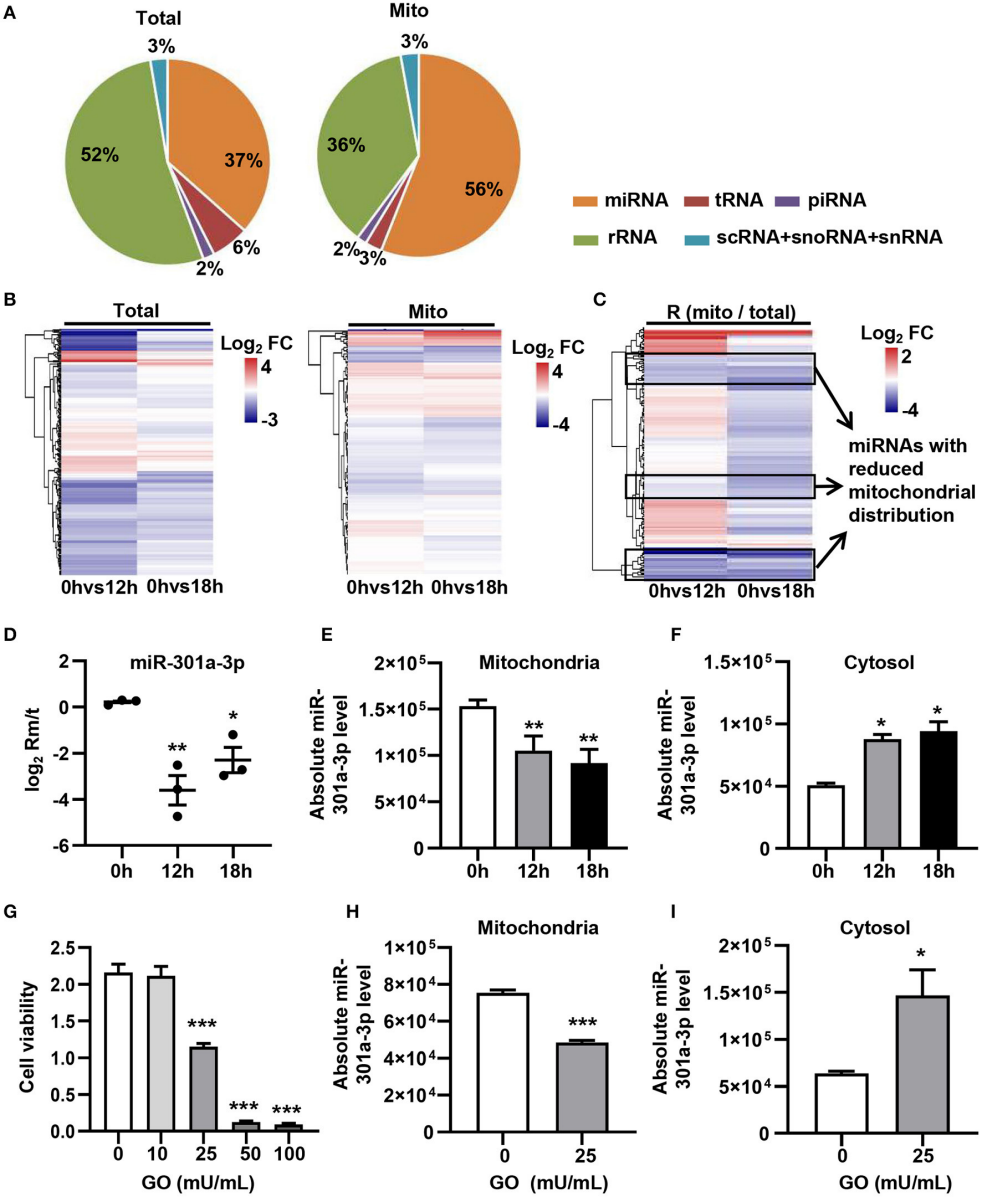
ROS redistributes miR-301a-3p between the cytosol and mitochondria. **(A)** The average frequency distribution of various non-coding RNA species identified in the whole cells (Total) and mitochondria (Mito) of HUVECs by small RNA sequencing. **(B)** Heatmap of the log_2_ fold changes in miRNA levels identified in mitochondria (Mito) and whole cells (Total) between H_2_O_2_-treated HUVECs at 0, 12, or 18 h. **(C)** Heatmap of the log_2_ fold changes in the relative mitochondrial distribution (Rm/t, ratio of the levels of miRNA in mitochondria to those in the whole cells) of miRNAs between HUVECs treated with H_2_O_2_ for 0, 12, or 18 h. **(D)** qRT-PCR analysis of the relative mitochondrial distribution (log_2_ Rm/t) of miR-301a-3p in HUVECs treated with H_2_O_2_ for 0, 12, and 18 h. **(E,F)** Absolute quantification of miR-301a-3p levels in mitochondria **(E)** and cytosol **(F)** of the H_2_O_2_-treated HUVECs for 0, 12, and 18 h. **(G)** The viability of HUVECs treated with glucose oxidase (GO) at different concentrations for 24 h estimated by MTT assay. **(H,I)** Absolute quantification of miR-301a-3p levels in mitochondria **(H)** and cytosol **(I)** of HUVECs treated with 25 mU/mL GO for 24 h. One-way analysis of variance (ANOVA) was used for **(D–G)**. Two-tailed Student's *t*-test was used for statistical analysis in H and I. Data are presented as mean ± SEM. *P < 0.05; **P < 0.01; ***P < 0.001.

In order to evaluate the dynamic changes in the distribution of miRNAs in mitochondria during endothelial injury, HUVECs were treated with 100 μM H_2_O_2_ for 12 and 18 h. Then, the miRNA profiling of mitochondria and the whole fraction of HUVECs was detected using small RNA sequencing. As shown in Figure 1, H_2_O_2_ impaired HUVECs by promoting apoptosis and inhibiting proliferation (Figure 1). To ensure the reliability of the sequencing results, the purity of mitochondria was evaluated at the protein and mRNA levels. The results showed that a mitochondrial protein, translocase of outer mitochondrial membrane 40 (Tomm40), was strongly enriched while two cytosolic proteins, β-actin and ribosomal protein S9 (RPS9) were undetected in the mitochondrial fraction (Supplementary Figure 1A). In addition, the ratios of mtRNAs (*MT-ND4* and *12S rRNA*) to cytosolic (*GAPDH, 18S rRNA*) or nuclear (*NEAT1*) RNAs in mitochondria were significantly higher than those in whole cells, which confirmed the high purity of the isolated mitochondria (Supplementary Figure 1B). Fifty-six percent of the mitochondria sequencing reads and 37% of the whole cell sequencing reads were aligned to miRNAs, respectively (Figure 2A). Total 193 high-abundance miRNAs (sequence reads > 100 in all samples) were detected in both mitochondrial and total fractions from H_2_O_2_-treated HUVECs for 0, 12, and 18 h. Compared with untreated HUVECs, the abundances of many miRNAs in total and mitochondrial fractions were significantly changed in H_2_O_2_-treated HUVECs (Figure 2B). To illustrate the dynamic distribution changes of miRNAs in mitochondria, the relative mitochondrial distributions (Rm/t, ratio of miRNA levels in mitochondria to those in whole cells) of these 193 miRNAs in each group were calculated. The results showed that H_2_O_2_ treatment significantly reduced the relative mitochondrial distribution of dozens of miRNAs in HUVECs (Figure 2C). Among them, miR-301a-3p was focused on because of its high abundance in mitochondria and the significant changes in its mitochondrial distribution. The results of qPCR further verified the decreased mitochondrial distribution of miR-301a-3p in HUVECs treated with H_2_O_2_ for 12 and 18 h (Figure 2D). Moreover, the level of miR-301a-3p in mitochondria and cytosol of HUVECs treated or untreated with H_2_O_2_ was absolutely quantified. The results demonstrated that the amount of miR-301a-3p was markedly decreased in the mitochondria, whereas it was significantly increased in the cytosol of H_2_O_2_-treated HUVECs (Figures 2E,F). To further validate the ROS-induced redistribution of miR-301a-3p between the cytosol and mitochondria, an alternative ROS-generating reagent, glucose oxidase (GO) (Mueller et al., 2009), was used to treat HUVECs. The results showed that GO could induce a concentration-dependent damage to HUVECs (Figure 2G). Compared to the untreated HUVECs, GO significantly decreased the level of miR-301a-3p in the mitochondria and increased its cytosolic level (Figures 2H,I). These results indicated that miR-301a-3p was redistributed between cytosol and mitochondria during ROS-induced endothelial cell injury.

### RNA-Binding Protein (RBP) MSI2 Specifically Binds to miR-301a-3p

**Figure 3:**
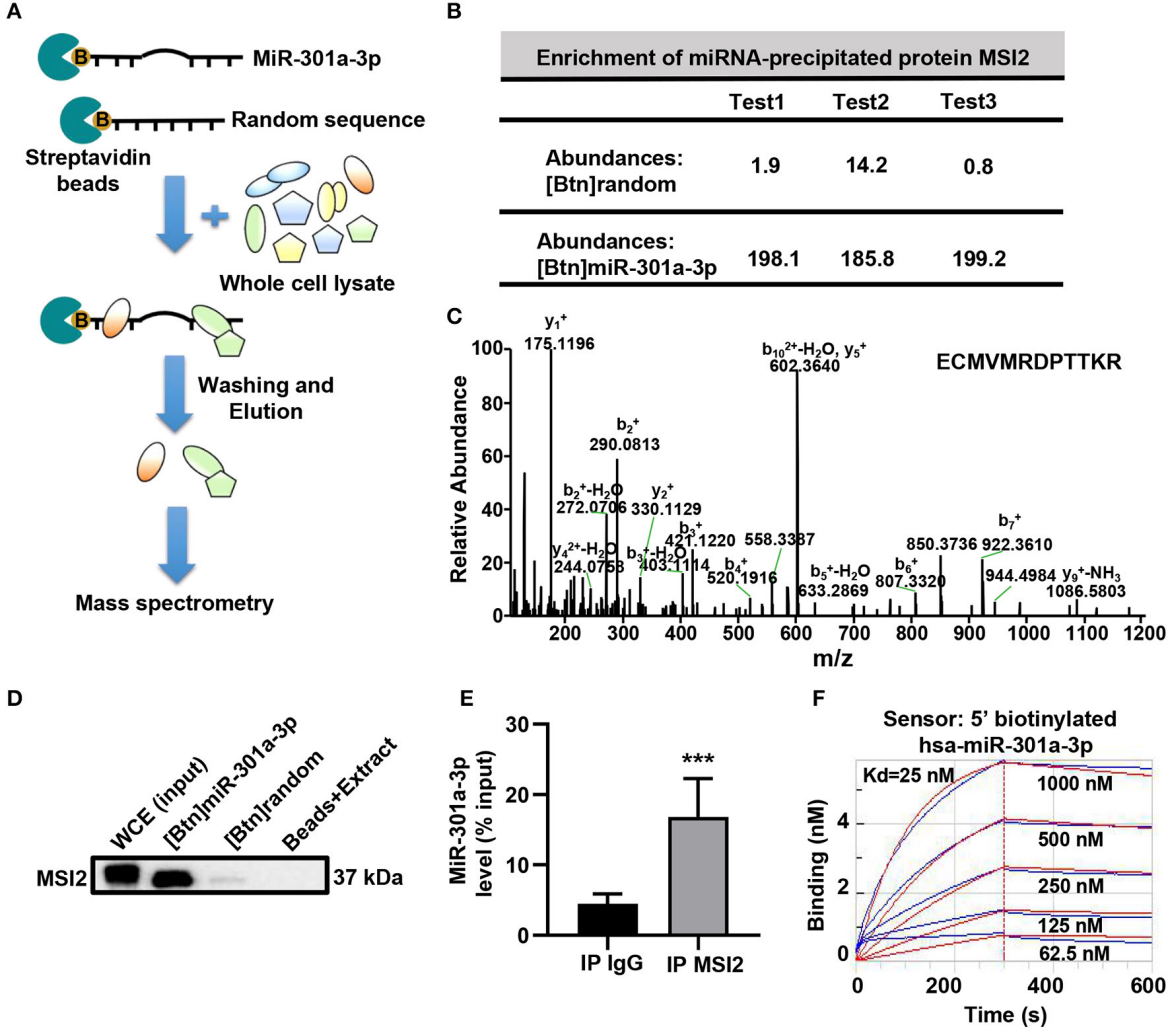
MSI2 binds to miR-301a-3p. **(A)** Schematic representation of biotinylated miRNA pull-down assay followed by label-free quantitative proteomic analysis. 5′biotinylated miR-301a-3p or random sequence (control group) was incubated with streptavidin beads and whole cell extracts of HUVECs. After washing and elution, the pull-down proteins were analyzed by MS/MS. **(B)** Relative abundances of MSI2 protein identified in three replicate experiments of label-free quantitative proteomic analysis for the product of biotinylated miR-301a-3p pull-down [(Btn) miR-301a-3p]. Biotinylated random small sequence [(Btn) random] was used as a negative control. **(C)** Representative MS/MS spectrum of a parent ion of MSI2 protein in the label-free quantitative proteomic analysis. The peptide sequence identified by this spectrum was shown in the upper right side. **(D)** Western blotting analysis of MSI2 protein in the product of the biotinylated miR-301a-3p pull-down assay. WCE: whole cell extracts; Beads+Extract: the mixture of streptavidin–agarose beads and whole cell extracts of HUVECs. **(E)** miR-301a-3p level in the immunoprecipitation products obtained by incubating anti-MSI2 antibody with the HUVECs lysates. The result was reported as percentage of the input sample (% input). The normal IgG was used as control (IP IgG). **(F)** The representative sensorgram of bio-layer interferometry (BLI) analysis for the binding kinetics of MSI2 and 5′ biotinylated miR-301a-3p. The blue curves represented the measured responses for each tested concentration of MSI2 protein. The overlapped red curves showed the global fitting results of the binding data. Wilcoxon rank-sum test was used for statistical analysis in **(E)**. Median with interquartile range was shown for **(E)**. ***P < 0.001.

To reveal the mechanism underlying the mitochondrial distribution of miR-301a-3p, biotinylated miRNA pull-down was performed (Figure 3A). To clearly distinguish between the non-specific and specific binding of proteins to miR-301a-3p, a label-free quantitative proteomic analysis was carried out to compare the relative amount of proteins bound to miR-301a-3p and a random sequence after biotinylated miRNA pull-down. Several RNA-binding proteins were identified to specifically bind to miR-301a-3p in endothelial cells (Supplementary Table 4). Among these proteins, MSI2 was the most enriched protein because its relative amount in miR-301a-3p group was over 122 times compared to that in the control group (random small sequence) (Figures 3B,C). Moreover, MSI2 has been reported to bind to pri-miR-7-1 (Choudhury et al., 2013), implying the possibility of MSI2 binding to miR-301a-3p and facilitating its transport to mitochondria. Therefore, MSI2 was further studied by western blot analysis. The results confirmed that MSI2 was enriched in the product of miR-301a-3p pull-down, whereas it was undetectable in the control group (Figure 3D). To further validate the endogenous interaction between MSI2 and miR-301a-3p, RNA immunoprecipitation analysis was carried out using anti-MSI2 or normal IgG antibodies. The results showed that the level of miR-301a-3p in immunoprecipitation products using anti-MSI2 antibody was significantly higher than that in immunoprecipitation products using normal IgG, demonstrating the interaction between endogenous MSI2 and miR-301a-3p (Figure 3E). Moreover, the biolayer interferometry assay, which can quantitatively measure the strength of interactions between biomolecules, also showed a very low equilibrium dissociation constant (Kd) of MSI2 and miR-301a-3p (25 nM), indicating a strong binding between them (Figure 3F). Therefore, these findings indicated that MSI2 specifically bound to miR-301a-3p in HUVECs.

### MSI2 Localizes in Mitochondria and Facilitates the Mitochondrial Distribution of miR-301a-3p

**Figure 4:**
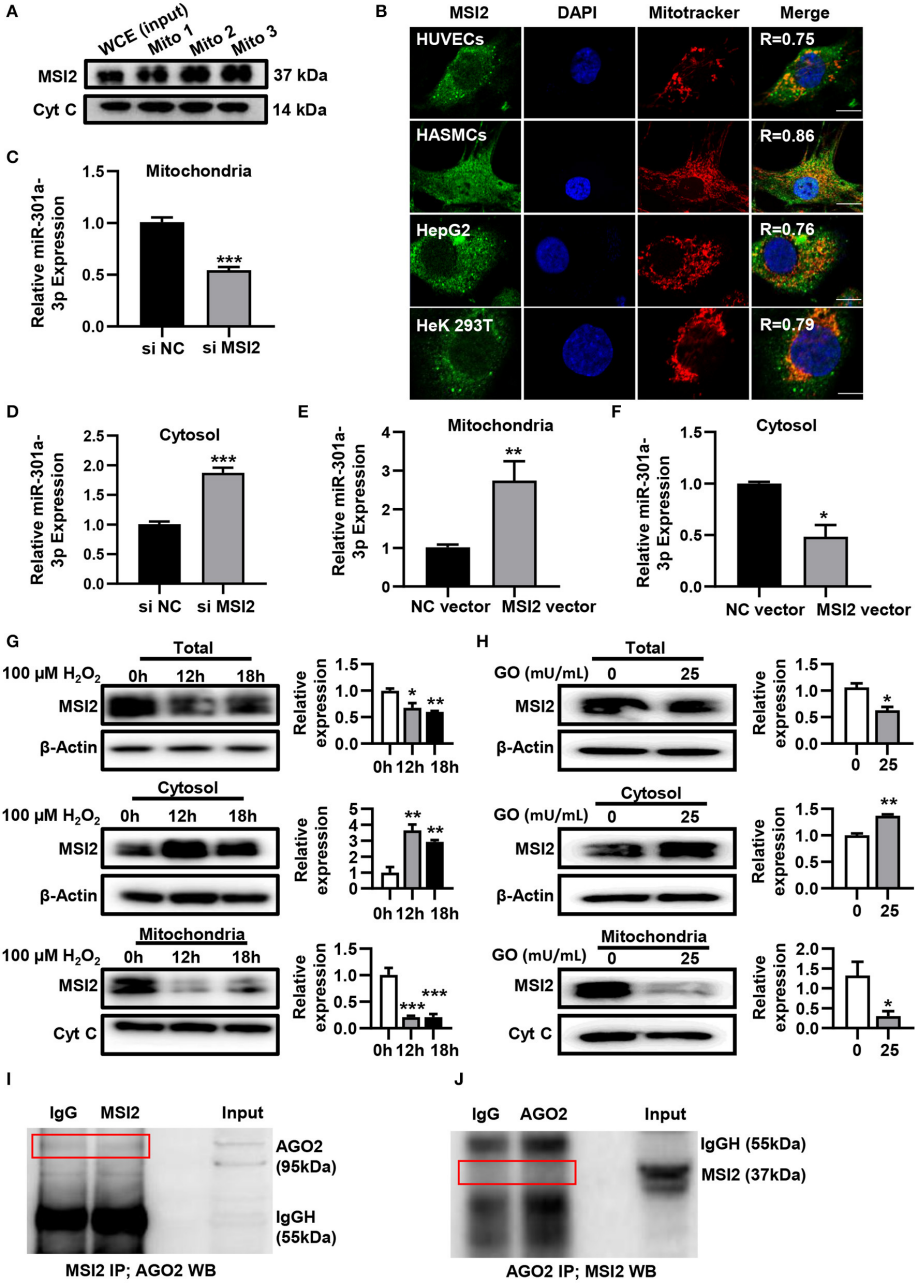
MSI2 exists in mitochondria and facilitates the distribution of miR-301a-3p in mitochondria. **(A)** Western blotting analysis of mitochondrial fractions of HUVECs for MSI2 and Cytochrome C. Mito: mitochondria; WCE: whole cell extracts. **(B)** Fluorescence colocalization of MSI2 and mitochondria in different types of cells. MSI2 was labeled with its Alexa Fluor 488-conjugated antibody (green). Mitochondria and nuclei were stained, respectively, with MitoTracker (red) and DAPI (blue). Yellow areas in the merged images represented the colocalization of MSI2 and mitochondria. R value represented the Mander's overlap coefficient between MSI2 and mitochondria calculated by Image-Pro Plus 6.0 software. Bar=10 μm. **(C,D)** The relative levels of miR-301a-3p in mitochondria **(C)** and cytosol **(D)** of HUVECs transfected with MSI2 siRNA compared to those in the control group. The 12S rRNA (mitochondria) and GAPDH (cytosol) were used as internal standards. **(E,F)** The relative levels of miR-301a-3p in mitochondria **(E)** and cytosol **(F)** of HUVECs transfected with MSI2 expression vector compared to those in the control group. **(G)** Western blotting analysis of MSI2 levels in the whole, cytosolic and mitochondrial fractions of HUVECs treated with H_2_O_2_ for 0, 12, and 18 h. **(H)** Western blotting analysis of MSI2 levels in the whole, cytosolic and mitochondrial fractions of HUVECs treated with GO for 24 h. **(I,J)** Immunoprecipitation analysis of the binding of Ago2 and MSI2 in HUVECs mitochondria. The immune complexes were formed by incubating mitochondrial lysates with anti-MSI2 (MSI2 IP) and then immunoblotted with anti-Ago2 antibody **(I)**, or by incubating mitochondrial lysates with anti-Ago2 (Ago2 IP) and then immunoblotted with anti-MSI2 antibody **(J)**. Mitochondrial lysates were used as input sample and normal IgG was used as the negative control (IgG). Two-tailed Student's *t*-test was used for statistical analysis in **(C–F,H)**. One-way analysis of variance (ANOVA) was used for **(G)**. Data are presented as mean ± SEM. *P < 0.05; **P < 0.01; ***P < 0.001.

To uncover the roles of MSI2 in the mitochondrial distribution of miRNAs, the localization of MSI2 in HUVECs was analyzed by immunoblot and immunofluorescence colocalization assays. The results of immunoblot showed that MSI2 protein was abundant in mitochondria of HUVECs and its amount was comparable to that of cytochrome c (Figure 4A). Immunofluorescence colocalization also demonstrated high overlap coefficients of MSI2 and mitochondria in a variety of cells, indicating that MSI2 was also present in the mitochondria of these cells (Figure 4B). From these results, it is evident that the abundant mitochondrial distribution of MSI2 is common and not a cell-type-specific phenomenon. To further investigate the role of MSI2 in miR-301a-3p mitochondrial distribution, HUVECs were transfected, respectively, with MSI2 siRNAs, MSI2 expression vector or corresponding negative control sequences (control group) (Supplementary Figures 1C,D). The results showed that, compared to control group, the level of miR-301a-3p in the mitochondria was significantly reduced while its cytosolic level was significantly increased in HUVECs transfected with MSI2 siRNAs (Figures 4C,D). Moreover, MSI2 overexpression increased the level of miR-301a-3p in the mitochondria and reduced its level in the cytosol (Figures 4E,F). These findings demonstrated that MSI2 could facilitate the mitochondrial distribution of miR-301a-3p. Additionally, H_2_O_2_ or GO treatment significantly decreased MSI2 levels in whole HUVECs and mitochondria but increased its cytosolic level, implying that MSI2 may be responsible for the H_2_O_2_- or GO-induced redistribution of miR-301a-3p in HUVECs (Figures 4G,H). Ago2 is an essential component of miRISC that has the ability to bind miRNAs, and has been detected in the mitochondria of many types of cells (Macgregor-Das and Das, 2018). Given the potential roles of Ago2 in mitochondrial distribution of miRNAs, Ago2 may interact with MSI2 in mitochondria. However, immunoprecipitation using the anti-Ago2 antibody or anti-MSI2 antibody in the mitochondrial pellet from HUVECs showed no interaction between Ago2 and MSI2 in mitochondria (Figures 4I,J). Together, the above results supported the conclusion that MSI2 could facilitate the distribution of miR-301a-3p in mitochondria.

## Discussion

Although several studies have established the subcellular location of miRNAs in the mitochondria (Latronico and Condorelli, 2012; Sripada et al., 2012b), the underlying mechanism is still unclear. Polynucleotide phosphorylase (PNPase) located in the mitochondrial intermembrane space was reported to facilitate the trafficking of miR-378 to the mitochondrial matrix. Overexpressing PNPase significantly increased miR-378 levels in the mitochondria of HL-1 cells (Shepherd et al., 2017). In the present study, we did not detect PNPase in the pull-down materials using biotinylated miR-301a-3p. This suggested that PNPase might not be involved in trafficking nucleus-encoded miR-301a-3p to mitochondria. We found that MSI2 could specifically bind to miR-301a-3p and facilitate its mitochondrial distribution. However, it is still unknown whether MSI2 and PNPase synergistically participate in miRNA mitochondrial distribution.

MSI2 has important roles in maintaining stem cell populations and regulating cancer initiation, progression, and metastasis (Kharas and Lengner, 2017). As an evolutionarily highly conserved RNA binding protein (RBP), MSI2 binds to many mRNAs involved in numerous oncogenic processes and regulates their stability and protein translation (Kudinov et al., 2017). Recent studies have linked certain RBPs to miRNA sub-cellular distribution. Human antigen R (HuR) was reported to regulate the extracellular distribution of multivesicular body (MVB)-associated miRNAs, whereas SYNCRIP and hnRNPA2B1 modulate miRNAs sorting in exosomes (Villarroya-Beltri et al., 2013; Mukherjee et al., 2016; Santangelo et al., 2016). However, the role of RBPs in mitochondrial distribution of miRNAs was not previously reported in the literature. In this study, we found that MSI2 silencing led to a significant decrease in miR-301a-3p levels in mitochondria of HUVECs, whereas MSI2 overexpression promoted the mitochondrial distribution of miR-301a-3p, which extended the physiological function of MSI2 to regulate the mitochondrial distribution of miRNA. A study found that a conserved three-nucleotide core motif UAG defined the RNA binding specificity of MSI2 (Zearfoss et al., 2014). Interestingly, this UAG motif is found in the sequence of miR-301a-3p, implying that MSI2 might bind to miR-301a-3p by recognizing this motif. Moreover, MSI2 may also bind to other miRNAs containing UAG in their sequences. In fact, we found that among the miRNAs that were redistributed between the mitochondria and cytosol induced by H_2_O_2_, some miRNAs apart from miR-301a-3p also contained UAG motif in their sequences, such as miR-103b and miR-10b-5p. The results of RNA immunoprecipitation with MSI2 antibody in HUVECs showed the high enrichment of miR-103b and miR-10b-5p in the immunoprecipitation products (Supplementary Figure 2). However, the molecular mechanisms underlying the binding of MSI2 to these miRNAs should be further studied.

Our findings demonstrated that H_2_O_2_ or GO significantly reduced the expression of MSI2. However, the mechanism by which ROS downregulates MSI2 is unclear. In fact, the current understanding of the upstream signaling pathways that regulate MSI2 expression is still very limited. It has been reported that Krüppel-like factor 4 (KLF4) transcriptionally inhibits MSI2 expression by directly binding to the MSI2 promoter in multiple pancreatic ductal adenocarcinoma (PDAC) cell lines (Guo et al., 2017). However, we found that H_2_O_2_ did not affect KLF4 expression in HUVECs, indicating that KLF4 might not be involved in the regulation of MSI2 by ROS (Supplementary Figure 3). Recently, ubiquitin carboxyl-terminal hydrolase 10 (USP10) was found to positively regulate MSI2 by post-transcriptional deubiquitination (Ouyang et al., 2019). Whether USP10 regulates the ubiquitination of MSI2 in our *in vitro* model should be further studied.

To the best of our knowledge, this study is the first to demonstrate that MSI2 interacts with miR-301a-3p and contributes to its distribution in mitochondria. Nevertheless, the detailed mechanism underlying the involvement of MSI2 in the mitochondrial distribution of miRNAs including miR-301a-3p is still unclear. However, the mechanisms by which many nucleus encoded RNAs apart from miRNAs are transported into the mitochondria have been studied. For example, 5S rRNA was reported to interact with the precursor of mitochondrial ribosomal protein L18 (MRPL18) in the cytosol, causing a conformational change in 5S rRNA that makes it recognized by Rhodanese and translocated into mitochondria (Smirnov et al., 2010, 2011). Obviously, it is conceivable that other proteins apart from MSI2 should also participate in miR-301a-3p mitochondrial import. In the pull-down materials using biotinylated miR-301a-3p, except for MSI2, we also found another interesting RBP, alpha-enolase. Enolase has been reported to be associated with the mitochondrial transport of yeast tRNA tRK1. The yeast tRNA tRK1 could bind to the cytosolic enolase (ENO2P) and the precursor of the mitochondrial lysyl-tRNA synthetase (preMSK or pre-LysRS) to form a complex on the mitochondrial membrane surface. Then, this complex was internalized into the mitochondrial matrix via the TOM/TIM protein transport system (Gowher et al., 2013). In addition, MSI2 has been demonstrated to interact with the translocase of inner mitochondrial membrane 10 homolog B (Huttlin et al., 2017), implying that MSI2-miRNA complex might be translocated into mitochondria via the mitochondrial transport system for proteins. Obviously, whether or not these above-mentioned proteins are involved in the mitochondrial transport of miR-301a-3p is an interesting issue that deserves to be investigated.

A growing body of evidence has suggested that the mitochondrial miRNA dynamic distribution is closely associated with the pathologies of many diseases (Borralho et al., 2014; Song et al., 2019). For example, miR-378 could redistribute into the interfibrillar mitochondria and regulate mitochondria encoded protein ATP6 in the diabetic heart (Jagannathan et al., 2015). Our study demonstrated that miR-301a-3p redistributed between the mitochondria and cytosol during the process of ROS-induced endothelial injury. It is well-known that endothelial injury plays an important role in the development of many cardiovascular diseases (Lerman and Zeiher, 2005). Moreover, miR-301a-3p has also been reported to induce HUVECs apoptosis and increase endothelial barrier permeability (Liu et al., 2020). Therefore, it might be assumed that the mitochondria-localized miR-301a-3p could rapidly re-enter into the cytosol and regulate its target genes, thus affecting endothelial function under oxidative stress conditions. In line with this assumption, the stored miRNAs in processing bodies have been evidenced to be delocalized and mediated translational repression when amino acid starvation occurs (Bhattacharyya et al., 2006). Nevertheless, more *in vivo* and *in vitro* studies are needed to investigate the pathological meaning of MSI2-mediated redistribution of miR-301a-3p in cardiovascular diseases.

## Data Availability Statement

The raw data supporting the conclusions of this article will be made available by the authors, without undue reservation.

## Author Contributions

QZ and ZW conceived the original idea and participated in experimental design. QG, XW, SZ, JG, XY, and XL performed the experiments. QG, QZ, and SZ analyzed the data. QG and QZ wrote the manuscript. ZW, LC, and XZ revised the manuscript. All authors contributed to the article and approved the submitted version.

## Conflict of Interest

The authors declare that the research was conducted in the absence of any commercial or financial relationships that could be construed as a potential conflict of interest.
