# Supplementary material for: RNA-Binding Protein MSI2 Binds to miR-301a-3p and Facilitates Its Distribution in Mitochondria of Endothelial Cells
Source: Front Mol Biosci. 2021 Jan 21;7:609828. doi: 10.3389/fmolb.2020.609828 (PMC7859344; doi:10.3389/fmolb.2020.609828)
Supplement: Supplementary file 1 [file Data_Sheet_1.docx]

Supplementary Material

## Supplementary Figures


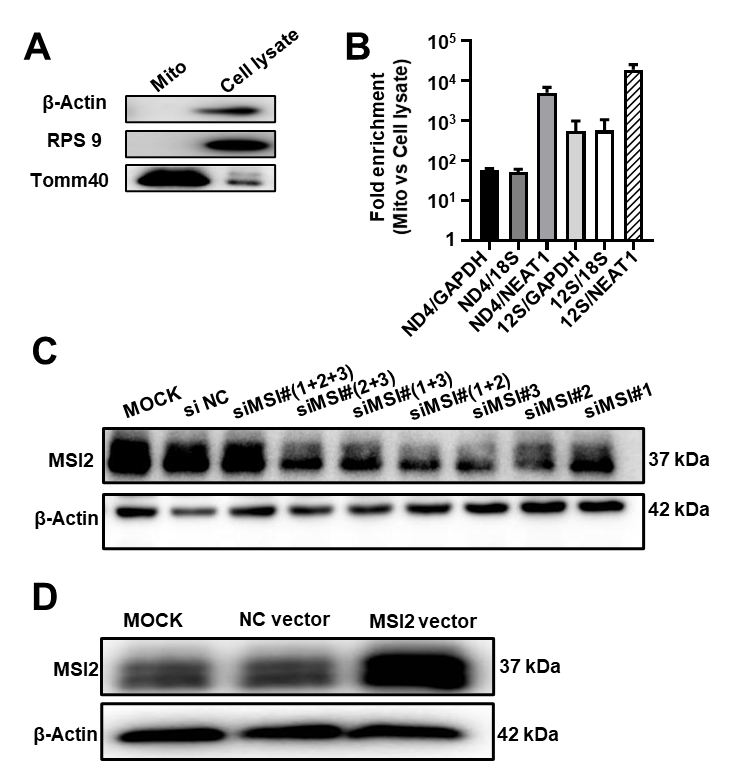


**Figure 1. (A)** Purity of mitochondria evaluated by western blotting. β-Actin and RPS9 were used as the cytosolic markers, Tomm40 was used as the mitochondrial marker. **(B)** qRT-PCR analysis of the ratios of mitochondrial RNAs (*ND4* and *12S rRNA*) to cytosolic (*GAPDH*, *18S rRNA*) and nuclear (*NEAT1*) RNAs in mitochondria compared to whole HUVECs. Data are presented as mean ± SEM. **(C)** MSI2 protein expression levels in HUVECs transfected with different MSI2 siRNAs and a scrambled sequence (negative control siRNA, si NC). MOCK: cellular extracts of untreated HUVECs. β-Actin was used as the loading control. The expression level of MSI2 protein was significantly downregulated by MSI2 siRNAs (si MSI2#3) in HUVECs. **(D)** MSI2 protein expression levels in HUVECs transfected with MSI2 expression vector and a control empty vector. MOCK: cellular extracts of untreated HUVECs. β-Actin was used as the loading control.


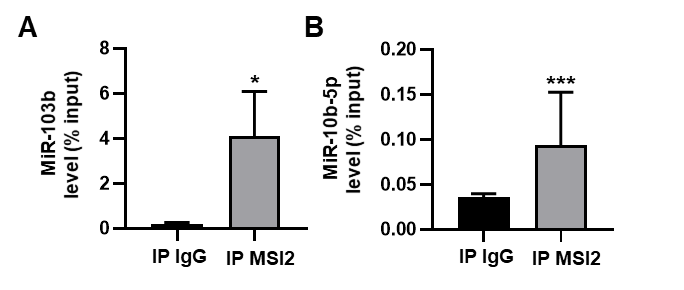


**Figure 2. Levels of miR-103b and miR-10b-5p in the immunoprecipitation products obtained by incubating anti-MSI2 antibody with the HUVECs lysates.** The results were reported as percentage of the input sample (% input). The normal IgG was used as control (IP IgG). Wilcoxon rank-sum test was used for statistical analysis. Median with interquartile range was shown. *: P < 0.05, ***: P <0.001.


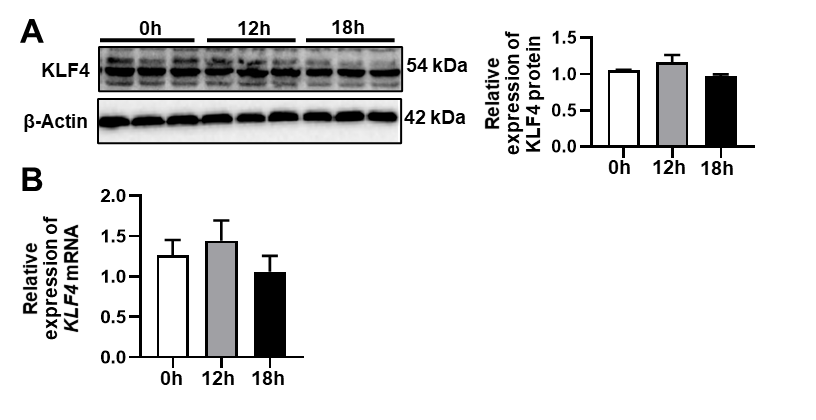


**Figure 3. The protein (A) and mRNA (B) levels of KLF4 in the HUVECs treated with 100 µM H_2_O_2_ for 0, 12, and 18 h analyzed by western blotting and qRT-PCR.** One-way analysis of variance (ANOVA) was used for statistical analysis. Data are presented as mean ± SEM.

## Supplementary Tables

**Table 1. Primary antibodies used in the present study.**

| Names | Company | Cat. No. | Concentration |
| --- | --- | --- | --- |
| Anti-β-Actin antibody | Proteintech | 60008-1-Ig | 1:1000(WB) |
| Anti-TOMM40 antibody | Abcam | ab185543 | 1:1000(WB) |
| Anti-Cytochrome C antibody | Abcam | ab133504 | 1:1000(WB) |
| Anti-MSI2 antibody | Proteintech | 10770-1-AP | 1:1000(WB) |
| Anti-RPS9 antibody | Abcam | ab157125 | 1:1000(WB) |
| Anti-AGO2 antibody | Proteintech | 10686-1-AP | 2ug (IP) |
| Anti-MSI2 antibody | Proteintech | 10770-1-AP | 2ug (IP) |
| Anti-MSI2 antibody | Proteintech | 10770-1-AP | 5ug (RIP) |
| Anti-KLF4 antibody | Abcam | ab215036 | 1:1000(WB) |

**Table 2.** **qRT-PCR primers for mRNAs and miRNAs used in the present study.**

| Genes |  | Primer sequences |
| --- | --- | --- |
| ND4 | Forward | 5'-TCGCTAACCTCGCCTTACC-3' |
|  | Reverse | 5'-GGAGAACGTGGTTACTAGCACA-3' |
| 12S rRNA | Forward | 5'-CACTACGAGCCACAGCTTAA -3' |
|  | Reverse | 5'-TCAGGGTTTGCTGAAGATGG -3' |
| 18S rRNA | Forward | 5'-GGCCCTGTAATTGGAATGAGTC-3' |
|  | Reverse | 5'-CCAAGATCCAACTACGAGCTT-3' |
| β-Actin | Forward | 5'-CATGTACGTTGCTATCCAGGC-3' |
|  | Reverse | 5'-CTCCTTAATGTCACGCACGAT-3' |
| GAPDH | Forward | 5'-GGAGCGAGATCCCTCCAAAAT-3' |
|  | Reverse | 5'-GGCTGTTGTCATACTTCTCATGG-3' |
| NEAT1 | Forward | 5'-CTTCCTCCCTTTAACTTATCCATTCAC-3' |
|  | Reverse | 5'-CTCTTCCTCCACCATTACCAACAATAC-3' |
| MSI2 | Forward | 5'-ACCTCACCAGATAGCCTTAGAG-3' |
|  | Reverse | 5'-AGCGTTTCGTAGTGGGATCTC-3' |
| KLF4 | Forward  Reverse | 5'-CCCACATGAAGCGACTTCCC-3'  5'-CAGGTCCAGGAGATCGTTGAA-3' |
| hsa-miR-301a-3p | MIMAT0000688 | 5'-GCCGCTAGTATTGTCAAAGCAAA-3' |
| hsa-miR-103b | MIMAT0007402 | 5'-CCCTCATAGCCCTGTACAAT-3' |
| hsa-miR-10b-5p | MIMAT0000254 | 5'-CCCTGTAGAACCGAATTTGTG-3' |

**Table 3.** **Biotinylated RNA oligonucleotides used in pull-down and BLI assays.**

| Name | Oligonucleotides sequence |
| --- | --- |
| Biotin-miR-301a-3p | 5'-CAGUGCAAUAGUAUUGUCAAAGC-3' |
| Biotin-random | 5'-CUUCAGUGACAGCACAUCGA-3' |

**Table 4. RBPs enriched in the biotinylated miR-301a-3p pull down materials.**

| **Accession** | **Description** | **Enrichment Fold** |
| --- | --- | --- |
| P31151 | Protein S100-A7 | 105.1 |
| Q96DH6-1 | RNA-binding protein Musashi homolog 2 | 122.1 |
| Q15434 | RNA-binding motif, single-stranded-interacting protein 2 | 55.5 |
| P07858 | Cathepsin B | 12.9 |
| P00441 | Superoxide dismutase [Cu-Zn] | 33.7 |
| Q13151 | Heterogeneous nuclear ribonucleoprotein A0 | 6.1 |
| Q92945 | Far upstream element-binding protein 2 | 23.9 |
| P05455 | Lupus La protein | 9.7 |
| P62937 | peptidyl-prolyl cis-trans isomerase A | 8.2 |
| Q9BUJ2-1 | Heterogeneous nuclear ribonucleoprotein U-like protein 1 | 5.4 |
| Q9BTM1-2 | Isoform 2 of Histone H2A.J | 3.5 |
| P39019 | 40S ribosomal protein S19 | 4.9 |
| Q6ZN17-1 | Protein lin-28 homolog B | 3.6 |
| P04406-1 | glyceraldehyde-3-phosphate dehydrogenase | 5.5 |
| P06733-1 | alpha-enolase | 22.3 |

## Supplementary Materials and Methods

### Protein digestion, peptide purification, nano liquid chromatography-tandem mass spectrometry (LC-MS/MS) analysis and protein identification and quantification

The proteins obtained from the biotinylated miRNA pull-down experiments were treated with dithiothreitol (DTT) and then alkylated with 1M iodoacetamide for 60 min at room temperature in the dark. Subsequently, the samples were centrifuged in Microcon YM-10 filter units (#1602002vs, Sartorius Stedim Biotech, Göttingen, Germany) to remove the detergent, DTT and other low-molecular-weight components. Next, the samples were washed with 25 mM NH_4_HCO_3_ (ABC buffer) and centrifuged at 14, 000 × *g*. The flow-through was discarded and the protein mixture was digested overnight at 37°C in the YM-10 filter unit using 1:25 (w/w) trypsin in ABC buffer. The digested samples were centrifuged and the flow-through was collected for MS analysis.

The MS analysis was performed on an EASY-nLC 1000 liquid chromatograph coupled with a Q Exactive Plus mass spectrometer (Thermo Scientific). A ReproSil-Pur C18-AQ trap column (5 μm, 0.2 mm × 35 mm) and a ReproSil-Pur C18-AQ analytical column (3 μm, 75 μm × 250 mm) were used to separate the peptides. The flow rate was 300 nL/min with mobile phase A (0.1% formic acid in 100% H_2_O) and B (0.1% formic acid in 100% acetonitrile). The gradient elution for mobile phase B was as follows: 2-7% from 0 to 4 min; 7-22% from 4 to 44 min; 22-35% from 44 to 54 min; 35-90% from 54 to 65 min. The mass spectrometer was operated in the positive ion mode. The data were acquired in the data-dependent mode with the “top 20” for collision-induced dissociation (CID)-MS2. The MS parameters were as follows: +2.1 kV spray voltage; 275°C capillary temp; 50% S-lens RF level; 350-1550 scan range; 35 000 resolution; AGC target, 3.0 x 10^6^ for MS survey and 1.0 x 10^5^ for MS2. maximum injection time, 100 ms for MS survey and 100 ms for MS2.

The label-free quantitative (LFQ) MS data analysis was performed using the Proteome Discoverer software 2.2 (Thermo Scientific). Briefly, the raw files were imported and searched against the UniProtKB/SwissProt database using Mascot (ver.2.3.0.2) (Matrix Science Ltd, London, UK). The SEQUEST HT parameters were as follows: trypsin enzyme, two missed cleavages allowed, minimum peptide length of 6, precursor mass tolerance of 4 ppm, and a fragment mass tolerance of 0.02 Da, carbamidomethylation of cysteine residues (+57.021 Da) as fixed modifications, oxidation of methionine residues (+15.995 Da) and deamidation of asparagine and glutamine (+0.984 Da) as variable modifications. Peptide identifications with strict false discovery rate (FDR) more than 1% were discarded.

### Bio-layer interferometry (BLI)

Bio-layer interferometry (BLI) technique was used to analyze the binding kinetics between MSI2 and miR-301a-3p by ForteBio Octet RED 96 (Forte Bio, Fremont, CA, USA). Briefly, the recombinant human MSI2 protein (167853, Abcam) was diluted to different concentrations (1000, 500, 250, 125, and 62.5 nM). The streptavidin-coated biosensors (Forte Bio, USA) were immobilized and saturated with 200 nM biotinylated miR-301a-3p oligonucleotides. Then, the biosensors were incubated with MSI2 protein for 5 min to measure the association. Next, the biosensors were transferred to PBS for 5 min to measure the dissociation. PBS buffer was used as the negative control. The equilibrium dissociation constants (Kd) defined as the ratio of k_off_ (dissociation) to k_on_ (association) rate constants were calculated using the curve fit (1:1) model in the Octet Origin software.
